# Endoscopic subserosal dissection of a giant gastric gastrointestinal stromal tumor with risk factors

**DOI:** 10.1055/a-2436-1353

**Published:** 2024-10-25

**Authors:** Joan Carles Balboa, Harold Benites-Goñi, Raquel Muñoz-González, Roman Turró, Merce Rosinach, Jorge Espinos, Hugo Uchima

**Affiliations:** 1 Endoscopy Unit, Teknon Medical Center, Barcelona, Spain; 2 Vicerrectorado de Investigación, Universidad San Ignacio de Loyola, Lima, Peru; 3 Endoscopy Unit, Gastroenterology Department, Hospital Universitari Germans Trias i Pujol, Badalona, Spain

Gastric gastrointestinal stromal tumors (GISTs) are mesenchymal tumors with variable malignancy potential. Standard management typically involves surgical resection, and owing to the low likelihood of regional lymph node metastasis, there is no need to perform dissection of clinically negative lymph nodes 1 . Advancements in endoscopic techniques and devices for closure of gastric perforation have enabled endoscopic resection to become a viable treatment option for gastric GISTs 2 , offering benefits such as faster recovery, shorter hospital stays, and reduced costs compared with laparoscopic resection 3 . Furthermore, endoscopic resection offers other advantages over surgical resection, including preservation of gastric functionality and reduced postoperative pain 4 .

An 87-year-old patient was referred to our unit with a predominantly intraluminal gastric GIST that had grown from 19 mm to more than 30 mm over 2 years ( Fig. 1 ), and which displayed necrotic foci on endoscopic ultrasound. The case was reviewed by a multidisciplinary committee, which decided to proceed with endoscopic resection, including intragastric fragmentation if necessary.

**Fig. 1:**
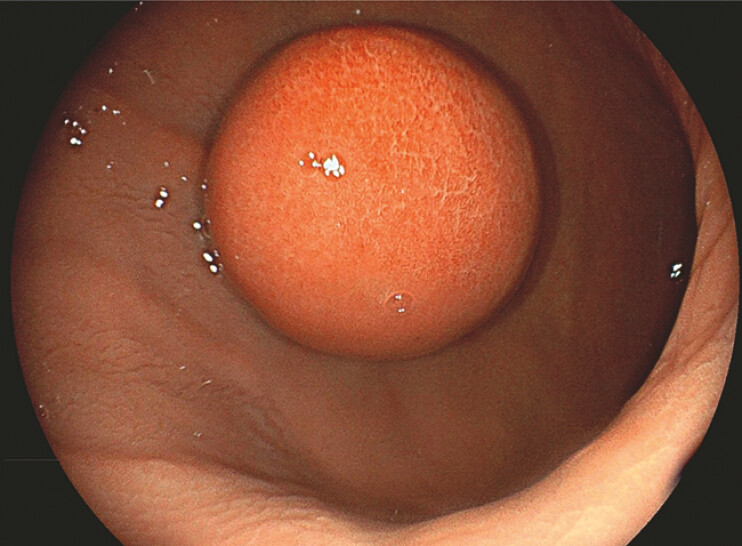
Intraluminal gastric gastrointestinal stromal tumor of almost 5 cm.

Endoscopic dissection of the GIST was conducted using a Splash M Knife (Pentax Medical, Tokyo, Japan) ( Video 1 ). Owing to the involvement of the muscularis propria ( Fig. 2 **a** ), a subserosal dissection was also performed ( Fig. 2 **b** ). Subsequent endoscopic suturing was completed using an Overstitch device (Apollo Endosurgery, Austin, Texas, USA) to close the full-thickness defect ( Fig. 2 **c** ). Given the resected specimen’s substantial size (5 cm), fragmentation of the sample was necessary to facilitate oral extraction and was carried out after the closure of the full-thickness defect ( Fig. 3 ).

Endoscopic submucosal and subserosal dissection of a giant gastric gastrointestinal stromal tumor with risk factors.Video 1

**Fig. 2:**
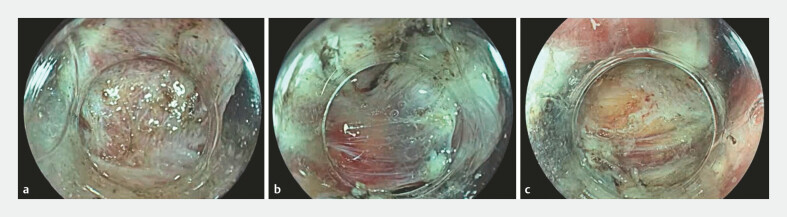
Endoscopy images. **a** Gastric gastrointestinal stromal tumor arising from the muscularis propria layer of the stomach wall. **b** Subserosal dissection preserving the serosal layer. **c** Preserved serosal layer.

**Fig. 3:**
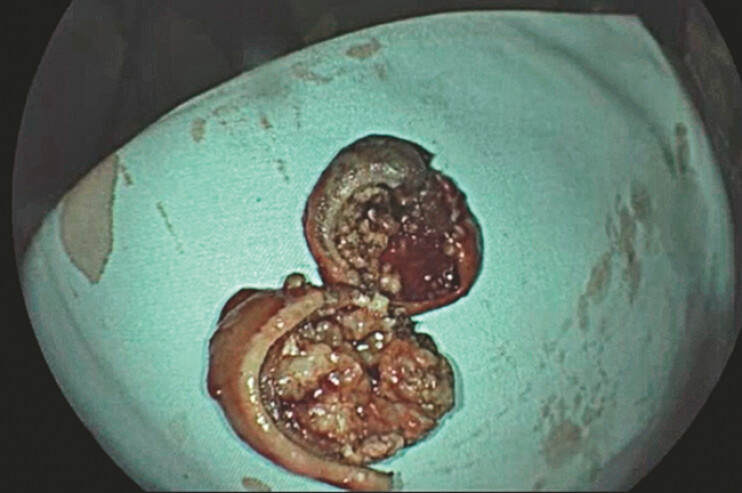
Fragments of the endoscopically resected gastric gastrointestinal stromal tumor.

The pathology report characterized the GIST as low risk, with fewer than 5 mitoses per 50 high-power fields and no necrotic foci. Follow-up gastroscopy and computed tomography scans at 12–18 months were normal.

In conclusion, endoscopic treatment of gastric GISTs larger than 3 cm is a viable alternative to surgery in selected cases. Fragmentation of the sample may be necessary for complete resection and extraction of larger lesions. Further studies are needed to establish standardized guidelines for the endoscopic management of large gastric GISTs.

Endoscopy_UCTN_Code_CPL_1AH_2AZ_3AD

## Notes

Endoscopy E-Videos https://eref.thieme.de/e-videos
*E-Videos* is an open access online section of the journal *Endoscopy* , reporting on interesting cases and new techniques in gastroenterological endoscopy. All papers include a high-quality video and are published with a Creative Commons CC-BY license. Endoscopy E-Videos qualify for HINARI discounts and waivers and eligibility is automatically checked during the submission process. We grant 100% waivers to articles whose corresponding authors are based in Group A countries and 50% waivers to those who are based in Group B countries as classified by Research4Life (see: https://www.research4life.org/access/eligibility/ ). This section has its own submission website at https://mc.manuscriptcentral.com/e-videos .

## References

[1] Casali PG Blay JY Abecassis N Gastrointestinal stromal tumours: ESMO–EURACAN–GENTURIS Clinical Practice Guidelines for diagnosis, treatment and follow-up Ann Oncol 2022 33 20 33 10.1016/j.annonc.2021.09.005 34560242

[2] Deprez PH Moons LMG OʼToole D Endoscopic management of subepithelial lesions including neuroendocrine neoplasms: European Society of Gastrointestinal Endoscopy (ESGE) Guideline Endoscopy 2022 54 412 429 10.1055/a-1751-5742 35180797

[3] Liu YB Liu XY Fang Y Comparison of safety and short-term outcomes between endoscopic and laparoscopic resections of gastric gastrointestinal stromal tumors with a diameter of 2–5 cm J Gastroenterol Hepatol 2022 37 1333 1341 35332574 10.1111/jgh.15834

[4] Abe N Takeuchi H Ohki A Comparison between endoscopic and laparoscopic removal of gastric submucosal tumor Dig Endosc 2018 30 7 16 29658656 10.1111/den.13010

